# Cross-sectional and longitudinal methods for describing the growth curve of Brahman females

**DOI:** 10.1007/s11250-026-05143-1

**Published:** 2026-06-15

**Authors:** Larissa Raffaela Trindade Borges, Felipe Augusto Fernandes, Alan Freire, Brennda Paula Gonçalves Araujo, Carlos Augusto Freitas Silva, Sarah Laguna Conceição Meirelles

**Affiliations:** 1 Faculdade de Zootecnia e Medicina Veterinária, Universidade Federal de Lavras Campus Universitário, Lavras, MG 37200-900 Brasil; 2 Instituto de Ciências Tecnológicas e Exatas, Universidade Federal do Triângulo Mineiro, Campus Univerdecidade, Uberaba, MG 38064-200 Brasil

**Keywords:** Body weight, Critical growth points, Genetic improvement, Nonlinear models

## Abstract

Growth curve modeling is a fundamental tool in animal production, allowing the analysis of the relationship between body weight and age through nonlinear statistical models. Traditionally, this curve has been obtained using the longitudinal method, which requires repeated measurements of the same individuals from birth to adulthood, making the process time-consuming and costly. As an alternative, the cross-sectional method uses single measurements from animals at different ages, reducing the time required for data collection. The present study aimed to evaluate the fit of different models to body weight data of Brahman females obtained by both longitudinal (*n* = 34) and cross-sectional (*n* = 219) methods, covering ages from birth to 65 months, select the most appropriate model, and compare the methods to assess the ability of the cross-sectional method to describe the growth curve of this population. In addition, critical points of the growth function were estimated to support decision-making in genetic improvement and productive management. The models were evaluated based on the coefficient of determination (R²), Akaike Information Criterion (AIC), and residual standard deviation, based on analyses performed in R software. The Gompertz model showed the best fit for the longitudinal data (R² = 0.9953), whereas the Logistic model provided the best fit for the cross-sectional data (R² = 0.9916). The results indicate that the cross-sectional method may represent a practical alternative to the longitudinal method for describing the growth curve of Brahman females, particularly when systematic data collection is not feasible.

**Supplementary Information:**

The online version contains supplementary material available at 10.1007/s11250-026-05143-1.

## Introduction

Beef production in tropical and subtropical regions is expected to increase substantially in the coming decades, reinforcing the need for efficient strategies to improve animal performance and production system sustainability (Cooke et al. 2020). In this context, Brahman cattle (Bos indicus), recognized for their adaptation to heat stress, play an important role in tropical production systems (Bessa et al. 2021; Mateescu et al. 2022). Because growth traits are directly associated with productivity, management efficiency, and genetic selection programs, accurate characterization of growth patterns is essential for optimizing production outcomes in this breed (Raidan et al. 2019).

Growth curve modeling is widely used in animal production to describe body development and support decisions related to management, nutrition, and breeding programs. Traditionally, growth curves are obtained using the longitudinal method, in which repeated measurements are collected throughout the animal’s life (Meigen 2022; Bonamy et al. 2020). Although considered the standard approach, the longitudinal method is costly, time-consuming, and often impractical under commercial cattle production conditions (Almeida et al. 2024). The cross-sectional method has been proposed as an alternative because growth trajectories are estimated from single measurements obtained from individuals of different ages within the same population, substantially reducing time and logistical constraints (Teixeira et al. 2021). While promising results have been reported in other species (Fernandes et al. 2022; Teixeira et al. 2021), direct comparisons between longitudinal and cross-sectional methods for modeling growth curves in Brahman females remain scarce, particularly under tropical production conditions. This limitation represents an important methodological gap regarding whether cross-sectional data can reliably reproduce growth patterns traditionally obtained from longitudinal designs.

Growth traits such as body weight and body measurements are economically important indicators of animal performance in Brahman cattle (Kamprasert et al. 2019), supporting breeding decisions and productivity improvement.

Therefore, the objectives of this study were to evaluate the suitability of the nonlinear models Brody, Gompertz, Logistic, and von Bertalanffy in describing the growth curve of Brahman females using both longitudinal and cross-sectional data collection methods; to compare these methods to determine the ability of the cross-sectional method to describe the growth curve of this population; and to analyze the critical points of the growth function, which provide practical information relevant to producers and genetic improvement programs.

## Materials and methods

### Animals and data

A database containing weight-age information for 219 Brahman female cattle from Fazenda Santa Éster da Casa Branca Agropastoril Ltda., located in Silvianópolis, Minas Gerais, Brazil, was used. Animals were weaned at approximately 7 months of age and were generally maintained on pasture, with supplementation provided during the dry season. Detailed information regarding management practices and nutritional strategies was not available in the database.

The data collection methods used were longitudinal and cross-sectional. For the longitudinal method, only animals with weight records for all time series were selected, using only 34 of the 219 available animals that met this criterion. The animals were divided into seven age classes (CIL), according to the frequency of data in each class, as follows: CIL zero: birth weight, CIL one: weight between one and ten months, CIL two: 11 to 20 months, CIL three: 21 to 30 months, CIL four: 31 to 40 months, CIL five: 41 to 50 months, CIL six: 51 to 60 months, and CIL seven: older than 60 months.

For the cross-sectional method, data from 219 animals were used, ranging from birth to 65 months of age. The animals were grouped into 14 age classes (CIT), also taking into account the frequency of data in each class: CIT zero: birth weight, CIT one: one to five months, CIT two: six to ten months, CIT three: 11 to 15 months, CIT four: 16 to 20 months, CIT five: 21 to 25 months, CIT six: 26 to 30 months, CIT seven: 31 to 35 months, CIT eight: 36 to 40 months, CIT nine: 41 to 45 months, CIT ten: 46 to 50 months, CIT eleven: 51 to 55 months, CIT twelve: 56 to 60 months, and CIT thirteen: 61 to 65 months.

The age class grouping was defined to balance biological relevance and data distribution across the growth trajectory, ensuring adequate representation of different developmental stages in both datasets. Although the longitudinal dataset comprised 34 animals, this sample size is considered adequate for nonlinear growth modeling with repeated measures and is consistent with, or exceeds, those reported in comparable studies Martins-Bessa et al. (2021); Afrouziyeh et al. (2021). It is important to note that assembling large longitudinal datasets in animal production systems is often constrained by operational costs and logistical limitations, particularly in resource-limited contexts.

Nevertheless, we recognize that, in the context of method comparison, the smaller size of the longitudinal dataset relative to the cross-sectional dataset may represent a limitation and should be considered when interpreting the results. Even so, the longitudinal data provide a consistent and informative basis for describing individual growth patterns, serving as a reference framework for evaluating the cross-sectional method’s ability to capture the overall growth trajectory.

### Non-linear models

The nonlinear models, Brody: $\:{y}_{i}=a\left(1-\left({exp}^{b-k{t}_{i}}\right)\right)+{\epsilon\:}_{i}$ (1), Gompertz: $\:{y}_{i}=a\times\:{exp}^{\left({-exp}^{k\left(b-{t}_{i}\right)}\right)}+{\epsilon\:}_{i}$ (2), Logistic: $\:{y}_{i}=\frac{a}{1+{exp}^{k\left(b-{t}_{i}\right)}}+{\epsilon\:}_{i}$ (3), and von Bertalanffy: $\:{y}_{i}=a\left(1-{\left(\frac{{exp}^{k(b-{t}_{i})}}{3}\right)}^{3}\right)+{\epsilon\:}_{i}$ (4), were used to fit the growth curve of the animals.

In Eqs. (1), (2), (3), and (4), the following are defined: “$\:{y}_{i}$” is the *i-th* observation of the response variable body weight, which can be obtained using either the cross-sectional or longitudinal method; “a” is the expected (asymptotic) value for adult weight; “b” is a location parameter associated with the inflection point of the model, i.e., the age at which the animal transitions from accelerated growth to decelerated growth until it stabilizes at the maximum value “a”; “k” represents the growth rate of the animal’s weight, or precocity index, as the higher the value of k, the less time is required for the animal to reach, for example, its adult weight; “ti” is the independent variable represented by the animal’s age in months; and “$\:{\epsilon\:}_{i}$” is the random experimental error associated with the i-th observation, assumed to be ${\varepsilon _i} \sim {N}(0,{\sigma ^2})$.

### Model fitting

The estimation of the parameters for these models was conducted using the least squares method, which allows for obtaining the System of Normal Equations (SNE) through the Gauss-Newton numerical algorithm. To assess the assumptions related to residual analysis, various statistical tests were performed. The Shapiro-Wilk test (1965) was applied to verify the normality assumption of the residuals, while the Durbin-Watson test (1971) assessed their independence. Additionally, the Breusch-Pagan test (1979) was used to investigate the homoscedasticity of the residual variances. All tests were performed with a 1% significance level, ensuring the robustness of the analysis and the model’s adequacy to the data.

The models were compared based on the quality of the fit provided, using the following criteria:

1. Residual standard deviation (RSD), calculated using the expression $\:RSD=\sqrt{MSE}$, where MSE is the mean square error. The smaller the RSD, the better the model fits.
2. Coefficient of determination (R²), obtained by: $\:{R}^{2}=1-\frac{SSE}{SSTotal}$, where SSE is the sum of squares of residuals and SSTotal is the total sum of squares. The coefficient of determination ranges from 0 to 1, with the best fit being the model whose R² is closest to 1.
3. Akaike Information Criterion (AIC), $\:\mathrm{AIC\:=\:-2\:log\:L\:(}{\uptheta\:}\mathrm{)\:+\:2\:(p)}$, where p is the number of model parameters and $\:\mathrm{L(}{\uptheta\:}\mathrm{)\:}$ is the natural logarithm of the maximum likelihood function. The smaller the AIC value, the better the model fit.

For all verifications, a 1% significance level was considered. After selecting the most appropriate model to describe the growth curves of Brahman females using both the longitudinal and cross-sectional methodologies, the growth curves and their derivatives from the models were plotted. Additionally, based on the parameter estimates of the most suitable model, a table with the obtained results was created.

The database organization was performed using SAS (Statistical Analysis System) software. The parameter estimates for the models, as well as the graphical adjustments, were obtained using the R statistical software/language (R Core Team, 2023).

### Identification of critical points of the growth curve

In the sigmoidal growth pattern (Figure of the fits), as the growth transitions from slow to faster, the maximum acceleration point (MAP) is identified. After this point, the characteristic under study reaches the inflection point (IP) and begins to decelerate until reaching the maximum deceleration point (MDP). Subsequently, when the equilibrium growth point is reached, where the individual has attained maturity, the point known as asymptotic deceleration (ADP) is reached (Teixeira et al. 2021). The IP corresponds to the value that nullifies the second derivative of the growth model, indicating the moment when the growth rate begins to decelerate, as seen in the work of Fernandes et al. (2022). The estimates of MAP, as well as MDP, can be determined by calculating the third derivative of the growth model, identifying its two roots. The ADP is one of the most relevant points in the growth curve. Located near the asymptote, it is interpreted as a stabilization point, representing the final phase of growth (Mischan et al. 2011). This point corresponds to the last root of the fourth derivative of the model, marking the final change in the curvature of acceleration. At this stage, the analyzed characteristic exhibits a slow acceleration that tends to zero.

The detailed calculation of the first to fourth-order derivatives of the Logistic and Gompertz models, which showed the best quality and fit evaluators, is presented in the Supplementary Material.

## Results

After parameter estimation, residual diagnostics were conducted for all fitted models within each method. There was no evidence of assumption violations (*p* > 0.01) for normality (Shapiro–Wilk), independence (Durbin–Watson), and homoscedasticity (Breusch–Pagan) for the models fitted under the cross-sectional method and for the Gompertz and von Bertalanffy models under the longitudinal method. The only exception was the Logistic model fitted under the longitudinal method, for which the Durbin–Watson test indicated residual autocorrelation (*p* < 0.01). To address this dependence structure, a first-order autoregressive process AR(1) was incorporated into the model. The parameter estimates for the von Bertalanffy, Logistic, Brody, and Gompertz models, for both the longitudinal and cross-sectional methods (Supplementary_Table_S1).

The comparative evaluation of nonlinear models by method revealed consistent differences in fit quality. Under the longitudinal method, the Gompertz model achieved the best performance (R² = 0.9953, AIC = 69.6249, RSD = 14.41; a = 579.60, k = 0.1010), outperforming the von Bertalanffy model, which ranked second (R² = 0.9897, AIC = 76.1227, RSD = 21.62). In contrast, for the cross-sectional method, the Logistic model provided the best fit (R² = 0.9916, AIC = 128.0984, RSD = 19.90; a = 581.28, k = 0.1800), followed by the Gompertz model (R² = 0.9875, AIC = 131.9868, RSD = 22.87).

**Fig. 1:**
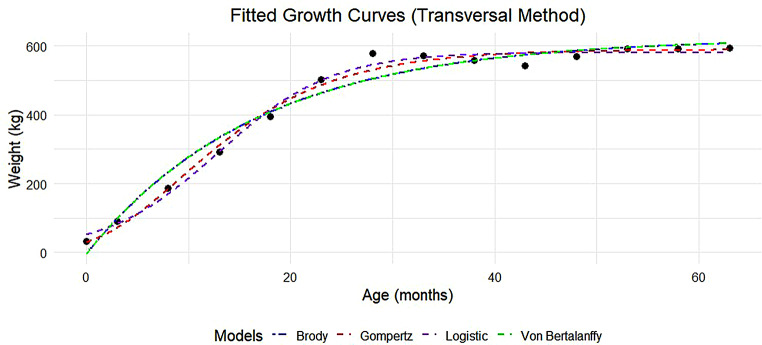
Body weights of Brahman female cattle as a function of age, determined using different nonlinear growth models, considering the cross-sectional method

**Fig. 2:**
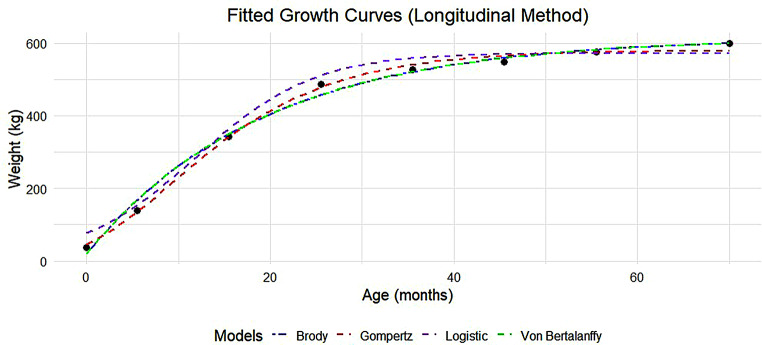
Body weights of Brahman female cattle as a function of age, determined using different nonlinear growth models, considering the longitudinal method

The point estimates of parameter a, when considered together with their standard errors, showed little variation between models across the different methods, which is particularly relevant given its practical importance as a key parameter for biological interpretation. By contrast, parameter k indicated a higher growth rate under the cross-sectional method, which may be attributed to the greater heterogeneity inherent to this approach, as it is based on measurements from different individuals rather than repeated observations of the same animals. Overall, the differences between the best and second-best models within each method were modest but consistent across goodness-of-fit criteria, supporting the robustness of model selection; these patterns are also reflected in the graphical fits (Figs. 1 and 2).

The detailed parameter estimates for the Gompertz and Logistic models show that the parameters obtained by the Gompertz model were slightly higher than those of the Logistic model (Supplementary_Table_S2).

### Derivatives and critical points

**Fig. 3:**
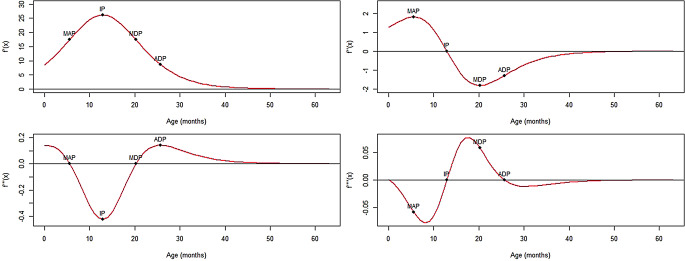
First (**a**), second (**b**), third (**c**), and fourth (**d**) order derivatives of the nonlinear Logistic model in describing the body weight growth curve of Brahman female cattle

**Fig. 4:**
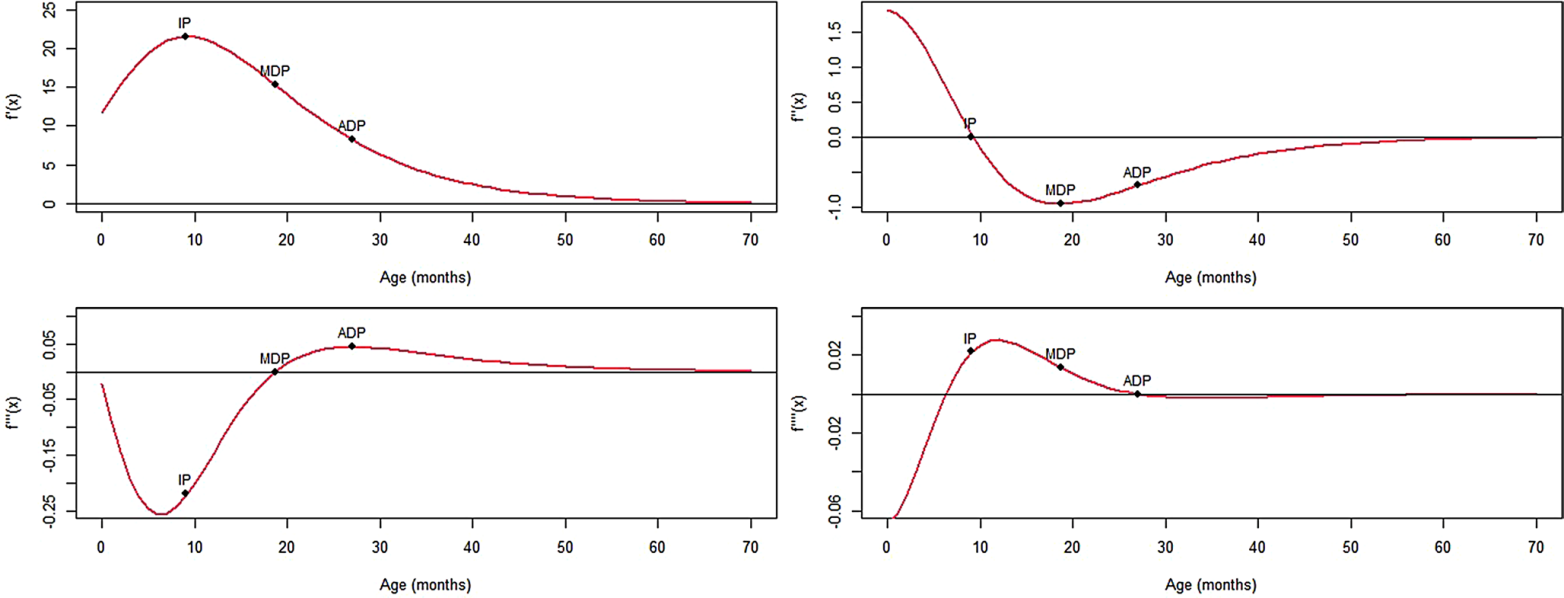
First (**a**), second (**b**), third (**c**), and fourth (**d**) order derivatives of the nonlinear Gompertz model in describing the body weight growth curve of Brahman female cattle

Figures 3 and 4 graphically display the behavior of the logistic and Gompertz models and the critical points found for each studied variable.

It can be observed in Figs. 3 and 4 the inflection point (IP), as well as other points such as the Maximum Acceleration Point (MAP), which demonstrates that in its positive phase, the acceleration function reaches its maximum value, after which it decreases, entering its negative phase and reaching a minimum point, known as the Maximum Deceleration Point (MDP) (Mischan and Pinho 2014).

After the Maximum Deceleration Point (MDP), negative acceleration progressively increases until reaching the last inflection point. From this point onward, deceleration begins to gradually decrease, approaching zero. At this final inflection point of acceleration, growth (y) reaches a condition of near stability, which characterizes the Asymptotic Deceleration Point (ADP), thus marking a potential point of growth cessation (Mischan and Pinho 2014).

## Discussion

The results observed in this study partially corroborate previous findings, which often point to the Brody model as more optimized for asymptotic weight projections, especially in breeds with prolonged growth (Lobo et al., 2003; Setâ et al. 2022), while the Gompertz model is effective in capturing nuances of acceleration and deceleration, particularly during growth phases (Gano et al. 2016). Discrepancies between the longitudinal and cross-sectional data collection methods in estimating growth parameters, such as the inflection point, reveal biological variability and methodological limitations. The higher sensitivity of k and inflection point estimates to the collection strategy suggests that growth dynamics are more affected by methodological choices than asymptotic body weight estimates. In this context, the cross-sectional method relies on single measurements, which may obscure individual differences influenced by environmental factors (Brandmaier et al. 2024; Parsons and McCormick 2024).

Such discrepancies have important biological implications for Brahman cattle and, more broadly, for zebu cattle. An earlier estimated inflection point in the longitudinal method may indicate an earlier onset of lean tissue deposition, reflecting greater metabolic efficiency and adaptability to nutritional management (Middleton et al. 2023). In contrast, the later inflection point estimated by the cross-sectional method may reflect greater between-animal heterogeneity and environmental variability (Fernandes et al. 2022). Despite this limitation, cross-sectional data may effectively approximate growth parameters when sufficiently large populations are evaluated (Middleton et al. 2023), which may explain the adequate performance of the Logistic model observed in this study, consistent with findings reported by Zimmermann et al. (2019). Likewise, the superior performance of the Gompertz model in describing early growth dynamics corroborates previous observations in Holstein Friesian cattle (Ratnasari et al. 2019), whereas studies in Angone cattle suggest that the Brody model may better represent asymptotic growth patterns (Setâ et al. 2022). Together, these findings reinforce that model suitability appears to depend not only on the data collection strategy, but also on breed-specific growth characteristics.

From a biological perspective, the growth phases identified by the models suggest distinct periods of nutritional and metabolic demand. During the initial growth stage (approximately 0–10 months), corresponding to the period of maximum acceleration (MAP), higher nutrient availability is required to support bone and muscle development, emphasizing the importance of adequate nutritional management during early growth (Silva et al. 2021). Around 10–20 months, near the inflection point (IP), growth rate reaches its maximum and animals may become more sensitive to nutritional restrictions, potentially affecting subsequent performance and feed efficiency (Harvey et al. 2021). Between 20 and 30 months, corresponding to the maximum deceleration period (MDP), growth begins to slow and metabolic costs associated with weight gain increase, indicating the need for adjustments in supplementation strategies to avoid excessive fat deposition (Mushfiq et al. 2023). After approximately 30 months, when the asymptotic deceleration phase (ADP) is reached, growth stabilizes and additional weight gain becomes progressively less efficient, suggesting reduced economic returns from maintaining animals for prolonged periods (Shevkhuzhev and Pogodaev 2022). These patterns indicate that the biological interpretation of growth curves may provide relevant information for optimizing nutritional interventions throughout different developmental stages.

Studies such as those by Untea et al. (2023) and Costa et al. (2021) highlight the importance of nutritional strategies tailored to inflection points identified by growth models, particularly in hot-climate breeds such as Brahman, which may require more intensive supplementation during early growth phases to achieve adequate development. In this context, the earlier inflection point estimated by the Gompertz model suggests the need for strategic nutritional support up to approximately 9.2 months of age, potentially maximizing feed efficiency without compromising adult weight.

Overall, the results indicate that the choice of growth model may be influenced by the data collection method adopted, meaning that the type of available data (longitudinal or cross-sectional), the modeling objective, and the growth characteristics of the studied population should all be considered. Although the Gompertz model showed superior performance under the longitudinal method, the Logistic model emerged as a valid and robust alternative under the cross-sectional method, particularly in situations where continuous growth monitoring is impractical and data collection must be performed at a single time point. These findings suggest that cross-sectional may represent a useful alternative in commercial production systems, where logistical limitations often restrict repeated measurements throughout the animal’s life. Future studies incorporating additional sources of variation, including genetic, nutritional, and environmental factors, may further improve the biological interpretation and applicability of growth models in Brahman cattle.

This study should be interpreted in light of several limitations. The longitudinal method was based on a relatively small sample, which, although adequate for nonlinear modeling with repeated measures and consistent with comparable studies, may limit population representativeness and the full characterization of between-animal variability in growth trajectories.

In turn, the cross-sectional method inherently incorporates a greater degree of unobserved heterogeneity, as animals evaluated at different ages may have been exposed to distinct environmental conditions, management practices, and nutritional regimes. At the same time, the larger sample size available for the cross-sectional method provides greater stability to parameter estimates and stronger empirical support for the observed variability, reducing the likelihood that such heterogeneity reflects sampling fluctuations alone. Nevertheless, the lack of detailed information regarding these factors may have contributed to additional variability and potential bias in parameter estimation, thereby influencing model performance.

Furthermore, the results are conditional on the assumptions underlying the adopted nonlinear models and the specified residual error structure. Although the diagnostic analyses did not indicate substantial violations, any degree of misspecification in the functional form or in the error structure may affect the robustness and interpretability of the findings.

Despite these limitations, some parameters showed greater stability across methods. In particular, parameter a, representing asymptotic body weight and having direct practical relevance, exhibited stable estimates across methods when accounting for associated standard errors, reinforcing its reliability for biological interpretation. In contrast, the comparatively higher estimates of k obtained under the cross-sectional method likely reflect the increased heterogeneity inherent to this approach and should therefore be interpreted with caution when deriving biological interpretations or management implications.

## Supplementary Information

Below is the link to the electronic supplementary material.

Supplementary Material 1

Supplementary Material 2

## Data Availability

The datasets generated and/or analyzed during the current study are available from the corresponding author on reasonable request.
