# Supplementary material for: Cross-sectional and longitudinal methods for describing the growth curve of Brahman females
Source: Trop Anim Health Prod. 2026 Jun 15;58(5):347. doi: 10.1007/s11250-026-05143-1 (PMC13269446; doi:10.1007/s11250-026-05143-1)
Supplement: Supplementary file 1 — Supplementary Material 1 [file 11250_2026_5143_MOESM1_ESM.docx]

**Supplementary Materials for Cross-sectional and longitudinal method for describing the growth curve of Brahman females**

**Derivatives of Growth Curves**

### Below are analytically presented the calculations of the first to fourth order derivatives for the Brody, Gompertz, Logistic, and von Bertalanffy models.

### 1. Derivatives of the Brody model

The Brody model is given by:

$$Y_{i}=\alpha[1-\beta e^{-(kx_{i})}]+\epsilon_{i}$$

To obtain the derivative of the model, the chain rule was successively applied at each order, which in this case is straightforward to understand.

The derivatives are:

$$Y'_{i}=\alpha\beta ke^{-(kx_{i})}$$

$$Y{''}_{i}=-\alpha\beta k^{2}e^{-(kx_{i})}$$

$$Y‴_{i}=\alpha\beta k^{3}e^{-(kx_{i})}$$

$$Y⁗_{i}=-\alpha\beta k^{4}e^{-(kx_{i})}$$

### 2. Derivatives of the Gompertz model

The Gompertz model is given by:

$$Y_{i}=\alpha e^{-e^{k(\beta-x_{i})}}+\epsilon_{i}$$

To obtain the derivatives up to the fourth order for this model, it was necessary to use the product rule.

The derivatives are:

$$Y'_{i}=\alpha ke^{-e^{k(\beta-x_{i})}}\cdot e^{k(\beta-x_{i})}$$

$$Y{''}_{i}=\alpha ke^{-e^{k(\beta-x_{i})}}\cdot(-e^{k(\beta-x_{i})})\cdot(-k)\cdot[e^{k(\beta-x_{i})}]+\alpha ke^{-e^{k(\beta-x_{i})}}\cdot[e^{k(\beta-x_{i})}\cdot(-k)]$$

The second order derivative simplified is:

$$Y{''}_{i}=\alpha k^{2}e^{-e^{k(\beta-x_{i})}}\cdot e^{2k(\beta-x_{i})}-\alpha k^{2}e^{-e^{k(\beta-x_{i})}}\cdot e^{k(\beta-x_{i})}$$

This is a more concise way to present the second order derivative:

$$Y{''}_{i}=k^{2}\alpha e^{-e^{k(\beta-x_{i})}}\cdot e^{k(\beta-x_{i})}\cdot[e^{k(\beta-x_{i})}-1]$$

Applying the product rule for the third derivative:

$$Y‴_{i}=(\alpha k^{2}e^{-e^{k(\beta-x_{i})}}\cdot(-e^{k(\beta-x_{i})})\cdot(-k)]e^{2k(\beta-x_{i})}+[\alpha k^{2}e^{-e^{k(\beta-x_{i})}}]\cdot(-2ke^{2k(\beta-x_{i})}))+([-\alpha k^{2}e^{-e^{k(\beta-x_{i})}}\cdot(-e^{k(\beta-x_{i})})\cdot(-k)]\cdot e^{k(\beta-x_{i})}+[\alpha k^{2}e^{-e^{k(\beta-x_{i})}}]\cdot e^{k(\beta-x_{i})}\cdot(-k))$$

Arranging and multiplying the entire previous equation:

$$Y‴_{i}=\alpha k^{3}e^{-e^{k(\beta-x_{i})}}e^{3k(\beta-x_{i})}-2\alpha k^{3}e^{-e^{k(\beta-x_{i})}}e^{2k(\beta-x_{i})}-\alpha k^{3}e^{-e^{k(\beta-x_{i})}}e^{2k(\beta-x_{i})}-\alpha k^{3}e^{-e^{k(\beta-x_{i})}}e^{k(\beta-x_{i})}$$

Simplifying the third order derivative:

$$Y‴_{i}=\alpha k^{3}e^{-e^{k(\beta-x_{i})}}e^{2k(\beta-x_{i})}\cdot[e^{k(\beta-x_{i})}-2]-\alpha k^{3}e^{-e^{k(\beta-x_{i})}}e^{k(\beta-x_{i})}\cdot[e^{k(\beta-x_{i})}+1]$$

The third order derivative can also be written as follows:

$$Y‴_{i}=\alpha k(ke^{k(\beta-x_{i})}-k)^{2}\cdot e^{k(\beta-x_{i})}-e^{k(\beta-x_{i})}-\alpha k^{3}e^{2k(\beta-x_{i})}-e^{k(\beta-x_{i})}$$

Applying the product rule to the previous equation to find the fourth order derivative:

$$Y⁗_{i}=(\alpha k^{3}e^{-e^{k(\beta-x_{i})}}\cdot(-e^{k(\beta-x_{i})})\cdot(-k)]\cdot e^{3k(\beta-x_{i})}+[\alpha k^{3}e^{-e^{k(\beta-x_{i})}}]\cdot(-3ke^{3k(\beta-x_{i})}))+([2\alpha k^{3}e^{-e^{k(\beta-x_{i})}}\cdot(-e^{k(\beta-x_{i})})\cdot(-k)]\cdot e^{2k(\beta-x_{i})}+[2\alpha k^{3}e^{-e^{k(\beta-x_{i})}}]\cdot(-2ke^{2k(\beta-x_{i})}))+([-\alpha k^{3}e^{-e^{k(\beta-x_{i})}}\cdot(-e^{k(\beta-x_{i})})\cdot(-k)]\cdot e^{2k(\beta-x_{i})}+[\alpha k^{3}e^{-e^{k(\beta-x_{i})}}]\cdot(-2ke^{2k(\beta-x_{i})}))+([-\alpha k^{3}e^{-e^{k(\beta-x_{i})}}\cdot(-e^{k(\beta-x_{i})})\cdot(-k)]\cdot e^{k(\beta-x_{i})}+[\alpha k^{3}e^{-e^{k(\beta-x_{i})}}]\cdot e^{k(\beta-x_{i})}\cdot(-k))$$

Arranging and multiplying the entire equation:

$$Y⁗_{i}=\alpha k^{4}e^{-e^{k(\beta-x_{i})}}e^{4k(\beta-x_{i})}-3\alpha k^{4}e^{-e^{k(\beta-x_{i})}}e^{3k(\beta-x_{i})}+2\alpha k^{4}e^{-e^{k(\beta-x_{i})}}e^{3k(\beta-x_{i})}-4\alpha k^{4}e^{-e^{k(\beta-x_{i})}}e^{2k(\beta-x_{i})}-\alpha k^{4}e^{-e^{k(\beta-x_{i})}}e^{3k(\beta-x_{i})}-2\alpha k^{4}e^{-e^{k(\beta-x_{i})}}e^{2k(\beta-x_{i})}-\alpha k^{4}e^{-e^{k(\beta-x_{i})}}e^{2k(\beta-x_{i})}-\alpha k^{4}e^{-e^{k(\beta-x_{i})}}e^{k(\beta-x_{i})}$$

Simplifying the fourth order derivative:

$$Y⁗_{i}=(\alpha k^{4}e^{-e^{k(\beta-x_{i})}}e^{3k(\beta-x_{i})})\cdot(e^{k(\beta-x_{i})}-3)+2\alpha k^{4}e^{-e^{k(\beta-x_{i})}}e^{2k(\beta-x_{i})}\cdot(e^{k(\beta-x_{i})}-2)-\alpha k^{4}e^{-e^{k(\beta-x_{i})}}e^{2k(\beta-x_{i})}\cdot(e^{k(\beta-x_{i})}-2)-\alpha k^{4}e^{-e^{k(\beta-x_{i})}}e^{k(\beta-x_{i})}\cdot(e^{k(\beta-x_{i})}-1)$$

The fourth order derivative can also be written in this way:

$$Y⁗_{i}=-2\alpha k^{3}(ke^{k(\beta-x_{i})}-k)\cdot e^{2k(\beta-x_{i})}-e^{k(\beta-x_{i})}-\alpha k^{3}(ke^{k(\beta-x_{i})}-2k)\cdot e^{2k(\beta-x_{i})}-e^{k(\beta-x_{i})}+\alpha k(e^{k(\beta-x_{i})}-k)^{3}\cdot e^{k(\beta-x_{i})}-e^{k(\beta-x_{i})}$$

### 3. Derivatives of the Logistic model

To obtain the first order derivative of the model, the quotient rule was used:

$$Y_{i}=\frac{\alpha}{1+e^{k(\beta-x_{i})}}+\epsilon_{i}$$

$$Y_{i}'=\frac{-\alpha e^{k(\beta-x_{i})}\cdot(-k)}{[1+e^{k(\beta-x_{i})}]^{2}}$$

$$Y_{i}'=\frac{\alpha ke^{k(\beta-x_{i})}}{[1+e^{k(\beta-x_{i})}]^{2}}$$

In this case, in addition to using the quotient rule, the chain rule was also applied.

$$Y_{i}''=\frac{(\alpha ke^{k(\beta-x_{i})}\cdot(-k))(1+e^{k(\beta-x_{i})})^{2}-(\alpha ke^{k(\beta-x_{i})})2(1+e^{k(\beta-x_{i})})e^{k(\beta-x_{i})}\cdot(-k)}{(1+e^{k(\beta-x_{i})})^{4}}$$

Multiplying and arranging the entire equation:

$$Y_{i}''=\frac{-\alpha k^{2}e^{k(\beta-x_{i})}(1+e^{k(\beta-x_{i})})^{2}}{(1+e^{k(\beta-x_{i})})^{4}}+\frac{2\alpha k^{2}e^{2k(\beta-x_{i})}(1+e^{k(\beta-x_{i})})}{(1+e^{k(\beta-x_{i})})^{4}}$$

Obtaining the final simplified second order derivative:

$$Y_{i}''=\frac{2\alpha k^{2}e^{2k(\beta-x_{i})}}{(e^{k(\beta-x_{i})}+1)^{3}}-\frac{\alpha k^{2}e^{k(\beta-x_{i})}}{(e^{k(\beta-x_{i})}+1)^{2}}$$

Applying the quotient rule in the equation for the third derivative:

$$Y_{i}^{‴}=\frac{(-\alpha k^{2}e^{k(\beta-x_{i})}\cdot(-k))((1+e^{k(\beta-x_{i})})^{2})-(-\alpha k^{2}e^{k(\beta-x_{i})})\cdot2(1+e^{k(\beta-x_{i})})e^{k(\beta-x_{i})}\cdot(-k)}{(1+e^{k(\beta-x_{i})})^{4}}+\frac{(4(\alpha k^{2}e^{k(\beta-x_{i})})e^{k(\beta-x_{i})}\cdot(-k))(1+e^{k(\beta-x_{i})})^{3}-(2\alpha k^{2}e^{2k(\beta-x_{i})})\cdot3(1+e^{k(\beta-x_{i})})^{2}\cdot e^{k(\beta-x_{i})}\cdot(-k)}{(1+e^{k(\beta-x_{i})})^{6}}$$

Arranging the multiplication:

$$Y_{i}‴=\frac{\alpha k^{3}e^{k(\beta-x_{i})}(1+e^{k(\beta-x_{i})})^{2}}{(1+e^{k(\beta-x_{i})})^{4}}-\frac{2\alpha k^{3}e^{2k(\beta-x_{i})}(1+e^{k(\beta-x_{i})})}{(1+e^{k(\beta-x_{i})})^{4}}-\frac{4\alpha k^{3}e^{2k(\beta-x_{i})}(1+e^{k(\beta-x_{i})})^{3}}{(1+e^{k(\beta-x_{i})})^{6}}+\frac{6\alpha k^{3}e^{3k(\beta-x_{i})}(1+e^{k(\beta-x_{i})})^{2}}{(1+e^{k(\beta-x_{i})})^{6}}$$

Obtaining the final simplified third order derivative:

$$Y_{i}‴=\frac{6\alpha k^{3}e^{3k(\beta-x_{i})}}{(e^{k(\beta-x_{i})}+1)^{4}}-\frac{6\alpha k^{3}e^{2k(\beta-x_{i})}}{(e^{k(\beta-x_{i})}+1)^{3}}+\frac{\alpha k^{3}e^{k(\beta-x_{i})}}{(e^{k(\beta-x_{i})}+1)^{2}}$$

Applying the quotient rule to the entire equation for the fourth derivative:

$$Y_{i}⁗=\frac{(\alpha k^{3}e^{k(\beta-x_{i})}\cdot(-k))(1+e^{k(\beta-x_{i})})^{2}-(\alpha k^{3}e^{k(\beta-x_{i})})\cdot2(1+e^{k(\beta-x_{i})})\cdot e^{k(\beta-x_{i})}\cdot(-k)}{(1+e^{k(\beta-x_{i})})^{4}}-\frac{(12(\alpha k^{3}e^{k(\beta-x_{i})})\cdot e^{k(\beta-x_{i})}\cdot(-k))(1+e^{k(\beta-x_{i})})^{3}-(-6\alpha k^{3}e^{2k(\beta-x_{i})})(3(1+e^{k(\beta-x_{i})})^{2}e^{k(\beta-x_{i})}\cdot(-k))}{(1+e^{k(\beta-x_{i})})^{6}}+\frac{(18\alpha k^{3}e^{2k(\beta-x_{i})}\cdot e^{k(\beta-x_{i})}\cdot(-k))(1+e^{k(\beta-x_{i})})^{4}-(6\alpha k^{3}e^{3k(\beta-x_{i})})(4(1+e^{k(\beta-x_{i})})^{3})e^{k(\beta-x_{i})}\cdot(-k)}{(1+e^{k(\beta-x_{i})})^{8}}$$

Obtaining the final simplified fourth order derivative:

$$Y_{i}⁗=\frac{24\alpha k^{4}e^{4k(\beta-x_{i})}}{(e^{k(\beta-x_{i})}+1)^{5}}-\frac{36\alpha k^{4}e^{3k(\beta-x_{i})}}{(e^{k(\beta-x_{i})}+1)^{4}}+\frac{14\alpha k^{4}e^{2k(\beta-x_{i})}}{(e^{k(\beta-x_{i})}+1)^{3}}-\frac{\alpha k^{4}e^{k(\beta-x_{i})}}{(e^{k(\beta-x_{i})}+1)^{2}}$$

### 4. Derivatives of the von Bertalanffy model

The von Bertalanffy model is given by:

$$Y_{i}=\alpha\left( 1-\frac{e^{k(\beta-x_{i})}}{3} \right)^{3}+\epsilon_{i}$$

To obtain the first order derivative, the chain rule was used:

$$Y_{i}'=3\alpha\left( 1-\frac{e^{k(\beta-x_{i})}}{3} \right)^{2}\cdot\left( -\frac{e^{k(\beta-x_{i})}}{3} \right)\cdot(-k)$$

$$Y_{i}'=\alpha ke^{k(\beta-x_{i})}\left( 1-\frac{e^{k(\beta-x_{i})}}{3} \right)^{2}$$

To obtain the second order derivative, it was necessary to use the product rule.

$$Y_{i}''=\alpha ke^{k(\beta-x_{i})}\cdot(-k)\left( 1-\frac{e^{k(\beta-x_{i})}}{3} \right)^{2}+\alpha ke^{k(\beta-x_{i})}\cdot2\left( 1-\frac{e^{k(\beta-x_{i})}}{3} \right)\cdot\left( -\frac{e^{k(\beta-x_{i})}}{3} \right)\cdot(-k)$$

Arranging and multiplying the equations:

$$Y_{i}''=-\alpha k^{2}e^{k(\beta-x_{i})}\left( 1-\frac{e^{k(\beta-x_{i})}}{3} \right)^{2}+\frac{2\alpha k^{2}e^{2k(\beta-x_{i})}}{3}\left( 1-\frac{e^{k(\beta-x_{i})}}{3} \right)$$

Applying the product and chain rules to obtain the third order derivative:

$$Y_{i}‴=-\alpha k^{2}e^{k(\beta-x_{i})}\cdot(-k)\left( 1-\frac{e^{k(\beta-x_{i})}}{3} \right)^{2}-\alpha k^{2}e^{k(\beta-x_{i})}\cdot2\left( 1-\frac{e^{k(\beta-x_{i})}}{3} \right)\cdot\left( -\frac{e^{k(\beta-x_{i})}}{3} \right)\cdot(-k)+\frac{2\alpha k^{2}e^{2k(\beta-x_{i})}\cdot(-e^{k(\beta-x_{i})})\cdot(-k)}{3}$$

Arranging and multiplying the equations:

$$Y_{i}‴=\alpha k^{3}e^{k(\beta-x_{i})}\left( 1-\frac{e^{k(\beta-x_{i})}}{3} \right)^{2}-2\alpha k^{3}e^{2k(\beta-x_{i})}\left( 1-\frac{e^{k(\beta-x_{i})}}{3} \right)+\frac{2\alpha k^{3}e^{3k(\beta-x_{i})}}{9}$$

Applying the product and chain rules to obtain the fourth order derivative:

$$Y_{i}⁗=\alpha k^{3}e^{k(\beta-x_{i})}\cdot(-k)\left( 1-\frac{e^{k(\beta-x_{i})}}{3} \right)^{2}+\alpha k^{3}e^{k(\beta-x_{i})}\cdot2\left( 1-\frac{e^{k(\beta-x_{i})}}{3} \right)\cdot\left( -\frac{e^{k(\beta-x_{i})}}{3} \right)\cdot(-k)-2\alpha k^{3}e^{2k(\beta-x_{i})}\cdot(-2k)\left( 1-\frac{e^{k(\beta-x_{i})}}{3} \right)-2\alpha k^{3}e^{2k(\beta-x_{i})}\cdot\left( -\frac{e^{k(\beta-x_{i})}}{3} \right)\cdot(-k)+\frac{2\alpha k^{3}e^{3k(\beta-x_{i})}\cdot(-3k)}{9}$$

Arranging and multiplying the equations:

$$Y_{i}⁗=-\alpha k^{4}e^{k(\beta-x_{i})}\left( 1-\frac{e^{k(\beta-x_{i})}}{3} \right)^{2}+\frac{2\alpha k^{4}e^{2k(\beta-x_{i})}}{3}\left( 1-\frac{e^{k(\beta-x_{i})}}{3} \right)+4\alpha k^{4}e^{2k(\beta-x_{i})}\left( 1-\frac{e^{k(\beta-x_{i})}}{3} \right)-\frac{2\alpha k^{4}e^{3k(\beta-x_{i})}}{3}-\frac{6\alpha k^{4}e^{3k(\beta-x_{i})}}{9}$$

Noting that the last fraction in the equation can be simplified by:

$$\frac{6\alpha k^{4}e^{3k(\beta-x_{i})}}{9}=\frac{2\alpha k^{4}e^{3k(\beta-x_{i})}}{3}$$

Therefore, the last two equations for $Y_{i}⁗$ can be added together. Calculating the least common multiple yields the final result for the fourth derivative:

$$Y_{i}⁗=-\alpha k^{4}e^{k(\beta-x_{i})}\left( 1-\frac{e^{k(\beta-x_{i})}}{3} \right)^{2}+\frac{14\alpha k^{4}e^{2k(\beta-x_{i})}}{3}\left( 1-\frac{e^{k(\beta-x_{i})}}{3} \right)-\frac{4\alpha k^{4}e^{3k(\beta-x_{i})}}{3}$$
