# Supplementary material for: Cross-sectional and longitudinal methods for describing the growth curve of Brahman females
Source: Trop Anim Health Prod. 2026 Jun 15;58(5):347. doi: 10.1007/s11250-026-05143-1 (PMC13269446; doi:10.1007/s11250-026-05143-1)
Supplement: Supplementary file 2 — Supplementary Material 2 [file 11250_2026_5143_MOESM2_ESM.docx]

Table 1. Parameter estimates and quality criteria for the von Bertalanffy, Logistic, Brody, and Gompertz models for the longitudinal and cross-sectional methods.

|  | Longitudinal females (1%) | | | |  | Cross-sectional females (1%) | | | | |
| --- | --- | --- | --- | --- | --- | --- | --- | --- | --- | --- |
| Models | | DPR | R2 | AIC | Models | | DPR | R2 | AIC |  |
| von Bertalanffy | | 21,62 | 0,9897 | 76,1227 | von Bertalanffy | | 37,51 | 0,9654 | 145,8450 |  |
| Logistic (AR1) | | 31,77 | 0,9738 | 78,2775 | Logistic | | 19,90 | 0,9916 | 128,0984 |  |
| Brody | | 21,62 | 0,9897 | 76,1227 | Brody | | 37,51 | 0,9654 | 145,8450 |  |
| Gompertz | | 14,41 | 0,9953 | 69,6249 | Gompertz | | 22,87 | 0,9875 | 131,9868 |  |

DPR = Relative Standard Deviation; R² = coeficiente of determination; AIC: Akaike Information Criterion

Table 2. Parameter estimates, quality criteria, and influential points (in months) for the Gompertz and Logistic models.

| Parameters | Estimate | Standard Error | P-value | SW | DW | BP | MAP | IP | MDP | ADP |
| --- | --- | --- | --- | --- | --- | --- | --- | --- | --- | --- |
|  |  | Gompertz |  |  |  |  |  |  |  |  |
| a | 579,5969 | 9,0400 | < 0,01 | 0,7796 | 0,0180 | 0,1616 | - | 9,21 | 18,73 | 27,00 |
| k | 0,1010 | 0,0071 | < 0,01 |  |  |  |  |  |  |  |
| b | 9,2130 | 0,5177 | < 0,01 |  |  |  |  |  |  |  |
|  |  | Logístico |  |  |  |  |  |  |  |  |
| a | 581,2814 | 7,8498 | < 0,01 | 0,8980 | 0,0560 | 0,6774 | 5,57 | 12,90 | 20,23 | 25,63 |
| k | 0,1800 | 0,0139 | < 0,01 |  |  |  |  |  |  |  |
| b | 12,9043 | 0,4941 | < 0,01 |  |  |  |  |  |  |  |

a = adult weight; k = precocity index; b = inflection point; Standard error = standard error of the estimate; P-value = statistical significance level; SW = Shapiro-Wilk statistic; DW = Durbin-Watson statistic; BP = Breusch-Pagan statistic; MAP = Maximum Acceleration Point; IP = Inflection Point; MDP = Maximum Deceleration Point; ADP = Asymptotic Deceleration Point.
